# Stratified Whole Genome Linkage Analysis of Chiari Type I Malformation Implicates Known Klippel-Feil Syndrome Genes as Putative Disease Candidates

**DOI:** 10.1371/journal.pone.0061521

**Published:** 2013-04-19

**Authors:** Christina A. Markunas, Karen Soldano, Kaitlyn Dunlap, Heidi Cope, Edgar Asiimwe, Jeffrey Stajich, David Enterline, Gerald Grant, Herbert Fuchs, Simon G. Gregory, Allison E. Ashley-Koch

**Affiliations:** 1 Department of Medicine, Duke University Medical Center, Durham, North Carolina, United States of America; 2 Department of Radiology, Duke University Medical Center, Durham, North Carolina, United States of America; 3 Department of Surgery, Duke University Medical Center, Durham, North Carolina, United States of America; University of Texas MD Anderson Cancer Center, United States of America

## Abstract

Chiari Type I Malformation (CMI) is characterized by displacement of the cerebellar tonsils below the base of the skull, resulting in significant neurologic morbidity. Although multiple lines of evidence support a genetic contribution to disease, no genes have been identified. We therefore conducted the largest whole genome linkage screen to date using 367 individuals from 66 families with at least two individuals presenting with nonsyndromic CMI with or without syringomyelia. Initial findings across all 66 families showed minimal evidence for linkage due to suspected genetic heterogeneity. In order to improve power to localize susceptibility genes, stratified linkage analyses were performed using clinical criteria to differentiate families based on etiologic factors. Families were stratified on the presence or absence of clinical features associated with connective tissue disorders (CTDs) since CMI and CTDs frequently co-occur and it has been proposed that CMI patients with CTDs represent a distinct class of patients with a different underlying disease mechanism. Stratified linkage analyses resulted in a marked increase in evidence of linkage to multiple genomic regions consistent with reduced genetic heterogeneity. Of particular interest were two regions (Chr8, Max LOD = 3.04; Chr12, Max LOD = 2.09) identified within the subset of “CTD-negative” families, both of which harbor growth differentiation factors (GDF6, GDF3) implicated in the development of Klippel-Feil syndrome (KFS). Interestingly, roughly 3–5% of CMI patients are diagnosed with KFS. In order to investigate the possibility that CMI and KFS are allelic, GDF3 and GDF6 were sequenced leading to the identification of a previously known KFS missense mutation and potential regulatory variants in GDF6. This study has demonstrated the value of reducing genetic heterogeneity by clinical stratification implicating several convincing biological candidates and further supporting the hypothesis that multiple, distinct mechanisms are responsible for CMI.

## Introduction

Chiari Type I Malformation (CMI) is characterized by displacement of the cerebellar tonsils below the base of the skull and occurs with an estimated prevalence of less than one percent in the United States [1], [2]. Although magnetic resonance imaging (MRI) is considered the gold standard for diagnosis, no universally accepted diagnostic criteria exist. Patients are usually considered affected if one cerebellar tonsil is herniated 5 mm or more [3] or both tonsils are herniated 3 mm or more [4]. CMI patients exhibit a wide range of neurologic symptoms, including headaches, dizziness, difficulty sleeping, numbness/tingling of an upper extremity, fatigue, nausea, shortness of breath, blurred vision, among others [5]. Currently, the only treatment to alleviate symptoms for CMI is suboccipital decompression surgery to both expand the cranial base and re-establish normal cerebrospinal fluid (CSF) flow.

Although multiple mechanisms have been proposed for cerebellar tonsillar herniation, including cranial constriction, cranial settling, spinal cord tethering, intracranial hypertension, and intraspinal hypotension [6], “classical” CMI is generally hypothesized to occur through the “cranial constriction” mechanism. More specifically, “classical” CMI is thought to be caused by an underdeveloped occipital bone, resulting in a posterior fossa (PF) which is too small and shallow to accommodate the normal sized cerebellum [7], [8]. Herniation of the cerebellar tonsils and an upward shift of the tentorium are thought to occur secondarily [8]. In addition to the “cranial constriction” mechanism, accumulating evidence supports an association between connective tissue disorders (CTDs) and some occurrences of CMI [9]. Importantly, CMI patients diagnosed with CTDs may represent a distinct class of patients that can be grouped under the “cranial settling” mechanism where both the occipital bone and posterior cranial fossa volume are normal in size but occipitoatlantoaxial joint instability exists [6].

While no disease gene has been identified for CMI to date, several lines of evidence support a genetic contribution to disease in at least a subset of nonsyndromic cases. These include twin studies [1], [10], [11], [12], [13], [14], [15], [16], familial clustering [10], [14], [15], [17], [18], [19], [20], [21], [22], [23], [24], [25], [26], [27], [28], [29], [30], and co-segregation with known genetic syndromes or conditions commonly found as part of a genetic syndrome, including Ehlers-Danlos syndrome [9], [31], [32], [33], Marfan syndrome [9], [34], [35], [36], Klippel-Feil syndrome [23], [37], [38], [39], [40], [41], [42], [43], [44], [45], [46], [47], [48], growth hormone deficiency [45], [46], [49], [50], [51], [52], [53], [54], [55], craniosynostosis [56], [57], and Neurofibromatosis type I [58], [59]. Furthermore, in a study conducted by Milhorat and colleagues, it was reported that out of a cohort of 364 symptomatic patients, 43 (12%) had at least one close relative with CMI with or without syringomyelia or idiopathic syringomyelia [23]. Additionally, 72 patients (20%) were reported as having at least one close relative with a similar symptomology without an official CMI diagnosis. Despite evidence for a genetic component, genetic studies for CMI have been limited. Ascertainment for family studies has been hindered due to a relatively rare disease prevalence together with the small proportion of cases that are familial [23]. In addition, the ability to obtain MRIs on a large series of individuals for diagnostic purposes and lack of consistent disease criteria has led to increased phenotypic variability across patients resulting in phenotyping challenges. Only one whole genome linkage screen, but no genome wide association studies, has been published for CMI. Using 23 Caucasian multiplex families containing 67 sampled individuals affected with CMI with or without syringomyelia, Boyles, et al. conducted a whole genome linkage screen and identified significant evidence for linkage to regions on chromosomes 9 and 15 [17]. While this study took an important first step in trying to elucidate the genetic basis of CMI, the genetics of CMI is still very much unknown. Our limited understanding of the biological mechanism, lack of consistent diagnostic criteria, and complex etiology pose exciting challenges for studying the genetics of CMI.

One major challenge is the variability of clinical presentation within the CMI patient population. This clinical heterogeneity presents as differences with respect to the pattern and severity of symptoms, response to surgery, presence of associated conditions, age of onset, and the extent of tonsillar herniation. As CMI is thought to be influenced by multiple genetic and environmental factors, this clinical heterogeneity likely reflects in part an underlying genetic heterogeneity. While this can have substantial implications during the design stage of a genetic study, the selection of families that are genetically homogeneous is not straight forward. One approach is to stratify families using clinical features that may identify groups of families that share similar genetic risk factors. In other words, reducing phenotypic variability may lead to a reduction in genetic variability. Although the pool of candidate clinical features to use for stratification can be quite large, previous clinical associations observed with the disorder provide some insight into which features to select.

To address these issues, we performed the largest whole genome linkage screen to date using 367 individuals from 66 nonsyndromic CMI multiplex families. Based on the limited evidence for linkage using the complete collection of families, we performed a stratified whole genome linkage analysis using the presence or absence of CTD related conditions and successfully identified putative CMI susceptibility genes in the genetically more homogeneous strata.

## Materials and Methods

### Ethics Statement

All participating family members provided written informed consent for this study. If participants were minors, written consent was obtained from a parent or legal guardian for participants younger than 18 years of age. Participants between 12 and 17 years of age were asked to provide written assent. Written informed consent and assent, when applicable, were obtained by approved clinical staff. Consent forms were either discussed in person or were mailed and then discussed over the telephone. All participant interactions were logged in Progeny 8 (Delray Beach, FL), our clinical data collection software program. The original signed consent is maintained by the study and a copy was provided to participants. The consent form, procedure described above, and this study were specifically approved by the institutional review board of Duke University Medical Center.

### Study Population

Participants were ascertained across the United States primarily through self-referral in response to advertisements on the web (e.g. Duke Center for Human Genetics and GeneTests), mailings and/or presentations to patient support groups and physician referral. Families were enrolled in the current study if at least two sampled individuals were diagnosed with CMI with or without syringomyelia. Exclusion criteria included the following: 1) families with a positive family history of a known genetic syndrome (e.g. Ehlers-Danlos syndrome, Marfan syndrome, Klippel-Feil syndrome, Crouzon syndrome, Neurofibromatosis), 2) family history of spina bifida or tethered cord syndrome, and 3) individuals thought to have a secondary form of CMI, such as occurring due to a brain tumor. Although syndromic families formally diagnosed with hereditary CTDs were excluded from our genetic screen, many family members exhibited conditions such as hypermobility, mitral valve prolapse and scoliosis which are often associated with CTDs as described in further detail below. Blood samples were collected from affected individuals and all available connecting family members, regardless of affection status. Additionally, study participants completed a family and medical history telephone interview, responded to a detailed clinical questionnaire, and submitted release forms for medical records and pre-surgical MRIs. When possible, a diagnosis of CMI was determined based on MRI measurements in which affection status was defined as cerebellar tonsillar herniation of 3 mm or more for both tonsils or herniation of 5 mm or more for either tonsil (refer to Table 1 for MRI availability). MRI measurements were taken from pre-surgical T1-weighted brain MRIs. Herniation of the left and right tonsils was measured linearly from the tip of the cerebellar tonsils perpendicularly to the foramen magnum on a sagittal image to the left and right of the midline, respectively. All measurements were reviewed by a board certified neuroradiologist (D.E). In the event that appropriate pre-surgical MRIs were not available, affection status was based on medical records or patient report when that was the only source available. Detailed population characteristics are provided in Table 1.

**Table 1:** Population characteristics.

Description	No. Individuals	No. Families
Total	367^a^	66
Number of affected individuals/family	2.77±0.99 [2]–[6] b	
**Sex**		
Female	223	65
Male	144	61
**CMI**		
Affected	183	66
Female	124	60
Male	59	44
Unaffected/Uncertain	184	61
Female	99	51
Male	85	53
**Syringomyelia**		
Affected	50	41
CMI-Affected	47	40
Female	26	24
Male	21	19
CMI-Unaffected/Uncertain	3	3
Female	2	2
Male	1	1
Unaffected/Uncertain	317	65
CMI-Affected	136	62
Female	98	53
Male	38	32
CMI-Unaffected/Uncertain	181	60
Female	97	50
Male	84	53
**Posterior fossa decompression surgery** c		
Yes	91	57
No	53	38
Unknown	39	24
**MRI data**		
Available	126	50
CMI-Affected	95	49
CMI-Unaffected/Uncertain	31	21
Unavailable	241	65
CMI-Affected	88	52
CMI-Unaffected/Uncertain	153	54

a Only considered genotyped individuals after exclusions were applied (See Methods section for details).

b Mean +/− standard deviation [range].

c Only considered affected individuals.

Abbreviations: CMI: Chiari Malformation Type I; No.: number.

### Genotyping and Quality Control

Blood samples were collected from study participants in EDTA tubes and DNA was extracted using the AutoPure LS® DNA extraction kit with Puregene® system reagents (Qiagen, Valencia, CA). A small amount of DNA (0.3 µg) was run on a 0.8% agarose gel in order to assess quality and each sample was quantified using the Nanodrop (Wilmington, DE). In total, 436 individuals from 75 families were genotyped using Illumina Human610-Quad BeadChips (San Diego, CA) per the manufacturer’s instructions and chips were scanned using the Illumina iScan system (San Diego, CA). Due to the duration of ascertainment for this study, genotyping was performed in two separate batches (Batch 1∶234 individuals from 40 families; Batch 2∶202 individuals from 41 families). In addition to samples from study participants, replicate samples were included across sample plates and checked for mismatches. Specifically, two CMI family (1 male, 1 female) and two Centre d’Etude du Polymorphism Humain (CEPH) (1 male, 1 female) samples were included across three 96-well sample plates per batch in an alternating pattern.

Quality control (QC) procedures were performed to ensure high quality data were used for analysis. Initial quality assessment was performed separately for each batch using the Illumina GenomeStudio genotyping module (San Diego, CA). Single nucleotide polymorphism (SNP) data (N = 585497 combined across batches 1 and 2) quality were further assessed using PLINK v1.07 [60] to detect deviations from Hardy-Weinberg equilibrium (HWE; calculated using unaffected founders), estimate minor allele frequency (MAF; calculated using unaffected founders), and identify Mendelian errors (Parent-Parent-Child (P-P-C)). Parent-Child (P-C) errors were identified separately using custom scripts since PLINK does not examine trios with missing parents. Additional sample quality checks in PLINK included estimating pairwise identity by descent (IBD) in order to verify known relationships and check for cryptic relatedness (–genome; markers were pruned first), identifying Mendelian errors as described previously, calculating inbreeding coefficients (–het; markers were pruned first), performing a multidimensional scaling analysis in order to detect population stratification as different ethnicities could alter MAF estimates thus affecting the linkage analysis (1 individual per family used; –cluster –ppc 1e-4–mds-plot 2; markers were pruned first), and checking for sex discrepancies (–check-sex).

### Whole Genome Linkage Analysis

Power for the whole genome linkage study was determined using SIMLINK [61]. Family structures, disease and sample statuses were based on the CMI multiplex linkage families used in the screen and provided as input for the simulations (N_replicates_ = 1,000). Additional model parameters used for the simulations included: disease MAF of 0.001, marker MAF of 0.30, and an affecteds-only, low penetrance function (0, 0.001, 0.001).

All linkage analyses were performed using MERLIN 1.1.2 and MINX (MERLIN in X) [62] and allele frequencies were estimated using founders only for all analyses subsequently described. Since the underlying genetic model for CMI is unknown, both parametric (model dependent) and nonparametric (model free) linkage analyses were performed. For the parametric linkage analysis, an “affecteds only” low penetrance function was used (0, 0.001, 0.001) and a rare disease allele frequency of 0.001 was assumed. We performed an “affecteds-only” analysis because unaffected/unknown individuals will only contribute genotypic information, while affected individuals will contribute both phenotypic and genotypic information to the analysis. This approach protects against misclassification of non-penetrant individuals within the families. In addition to the standard LOD score analysis, MERLIN also provides estimates of the proportion of linked families (α) and the maximum heterogeneity LOD score (HLOD) which was used to detect linkage allowing for heterogeneity for the parametric analysis [62]. For the nonparametric linkage (NPL) analysis, the S_all_ scoring function was used which assesses IBD sharing across subsets of affected individuals [63]. In addition, both the Kong and Cox linear and exponential model were applied in order to evaluate statistical significance [64].

For both the parametric and nonparametric linkage analysis, two-point and multipoint analyses were performed. In order to maintain the correct type I error rate when conducting a multipoint analysis in families when one or both parents are missing, the option, “–rsq”, in MERLIN was implemented which allows for the modeling of inter-marker linkage disequilibrium (LD) between SNPs [62]. An r^2^ threshold of 0.16 [65] was selected to group SNPs into clusters.

Prior to whole genome linkage analysis, MERLIN’s error detection option was used to identify possible genotyping errors, such as unlikely double recombinants [62]. All genotypes flagged as potentially problematic were set as missing for the linkage analysis.

### Stratified Whole Genome Linkage Analysis

Families (N = 66) were stratified based on medical record documentation or self-reported family history of any of the following CTD related conditions: hypermobility (N = 4), kyphosis (N = 2), aneurysm (N = 11), mitral valve prolapse (N = 9), pectus excavatum (N = 1), scoliosis (N = 15), orthostatic hypotension (N = 1), supraventricular tachycardia (N = 2), heart valve disease (N = 12), and/or heart murmur (N = 6). In total, 34 families were grouped as “CTD-positive” and the remaining 32 families were “CTD-negative”. CTD-positive families had a significant history for one (47.1%), two (32.4%), three (14.7%), or five (5.9%) of the CTD-related conditions described above.

### Permutation Tests

A series of permutation tests were performed using custom scripts in order to determine genome-wide and chromosome-wide empirically derived significance levels for the stratified analyses conditional on the prior evidence for linkage. This was used to assess the relationship between the increased evidence for linkage and clinical criteria used to stratify families. For both the parametric (two-point and multipoint) and nonparametric (linear and exponential model; two-point and multipoint) analyses the following was performed: 1) The dataset was randomly split in half creating two datasets each containing 33 families, 2) Linkage analyses were conducted using MERLIN 1.1.2 and MINX for the X chromosome [62] as previously described in each set of families separately, 3) For each analysis (N = 6), the maximum LOD score was retained for each chromosome as well as genome-wide, and 4) Steps 1 through 3 were repeated 500 times in order to construct an empirical distribution (N_total_ = 1000).

### Candidate Gene Sequencing

Candidate gene selection for *de novo* sequencing was based on results from the CTD stratified whole genome linkage analysis described below. All affected individuals from any of the 66 linkage families that showed a positive family specific LOD score for the peak marker on chromosome 8 (rs2446871) or chromosome 12 (rs10505755) were selected for Sanger sequencing of growth differentiation factors, GDF6 and GDF3. In total, 96 affected individuals from 39 families and 75 affected individuals from 28 families were initially screened for mutations in GDF6 and GDF3, respectively. Seventeen GDF6 primer sets were designed to cover the exons (including intron-exon boundaries), 5′ and 3′ untranslated regions (UTR), as well as three intronic regions with high conservation (UCSC genome browser: Placental Mammal Basewise Conservation by PhyloP). Three GDF3 primer sets were designed to cover exons (including intron-exon boundaries) and 5′ and 3′ UTRs. Primer sequences, PCR conditions and kits are described in detail (Tables S1–S2). PCR amplicons and primers were sent off to Agencourt (Danvers, MA) and GeneWiz (South Plainfield, NJ) for Sanger sequencing. SNPs, as well as insertions and deletions (indels) were identified using Sequencher 5.0 (Ann Arbor, MI) and all sequences were manually inspected for each variant and indel called. Additionally, all individuals were checked for sufficient sequencing coverage for each amplicon. The nomenclature used to describe novel variants was based on recommendations by den Dunnen and Antonarakis [66]. Bi-directional sequencing in affected as well as unaffected family members was performed in order to follow-up eight identified variants that met subsequent criteria: 1) 1000 Genomes European MAF <0.05 (Integrated Phase 1 Release v3), 2) Identified in more than one affected individual (except two novel variants that were identified within the same family), and 3) 1000 Genomes European MAF was less than the study population MAF which was roughly estimated using all affected family members. Sequence data for novel variants were submitted to GenBank under accession numbers KC174775-KC174780.

## Results

### Genotyping Quality

Out of the 592532 SNPs genotyped on the Illumina Human610-Quad BeadChips (San Diego, CA), 7544 (1.3%) and 6835 (1.2%) SNPs were excluded from batches 1 and 2, respectively, due to call rates <98%, presence on chromosomes 24–26, high replicate error rate, as well as Illumina specific quality metrics including AB T Mean, AB R Mean, cluster separation, among others. Within each batch, replicate reproducibility rates exceeded 99.999% and all samples, except for one of the CEPH samples in batch 2, had a call rate >99%. Additional SNPs were excluded with Mendelian errors in >4% families (N = 220), MAF <0.05 (N = 66355), HWE p<0.001 (N = 275), identical physical location (Human genome build GRCh37/hg19; N = 2), no genetic distance available from deCODE (N = 948), call present in only batch 1 (N = 2445), call present in only batch 2 (N = 2991), and identical genetic position (based on two decimal places; N = 290918). Genotypes for all SNPs showing non-Mendelian inheritance were set as missing for the entire family. A total of 221343 SNPs remained after filtering and were used to construct the two-point linkage map. From those remaining SNPs, 12056 were selected for use in the multipoint linkage analysis using criteria such as genetic distance in order to create an evenly spaced map and high MAF estimates resulting in increased marker heterozygosity (Mean distance (cM) between SNPs: 0.31±0.008; Mean MAF: 0.42±0.09). In addition to SNP exclusions, three individuals were excluded due to large genomic duplications and/or regions of loss of heterozygosity detected from log R ratio and B allele frequency plots in Illumina GenomeStudio. This ultimately resulted in a total loss of 14 individuals due to two families that were no longer useful for linkage analysis. After additional sample exclusions were applied, 367 individuals from 66 families remained for analysis. Detailed sample exclusions are provided, along with the multidimensional scaling analysis used to identify sample outliers (Table S3 and Figure S1).

### Whole Genome Linkage Screen: Primary Analysis

SIMLINK [61] was used to estimate power for our whole genome linkage screen. Assuming homogeneity and a low recombination fraction (Θ = 0.01), the probability of obtaining a LOD score exceeding 3 was 0.94 using all 66 families collectively suggesting that we had adequate power to conduct the whole genome screen.

Following data quality assessment, both two-point and multipoint parametric and nonparametric linkage analyses were conducted. Initial findings across all 66 families showed minimal evidence for linkage, with no multipoint maximum LOD scores exceeding 2 although several two-point LOD scores exceeded 3 across the various models (See Table S4 for summary). Although no multipoint LOD scores exceeded 2, maximum multipoint LOD scores between 1.25 and 2 were found on 2q37.3 (Max LOD = 1.40, exponential model), 8q21.3–q22.2 (Max LOD = 1.38, linear model), 9p22.3–p21.3 (Max HLOD = 1.96, α = 0.28), 9q21.31–q22.33 (Max LOD = 1.32, linear model), 12p13.31–p13.2 (Max HLOD = 1.54, α = 0.25), and 18q21.33–q22.3 (Max HLOD = 1.78, α = 0.22). Based on the limited significance of these results, stratified analyses using clinical criteria were conducted in order to reduce potential genetic heterogeneity thus improving power to localize CMI susceptibility genes.

### Stratified Whole Genome Linkage Screen

**Figure 1:**
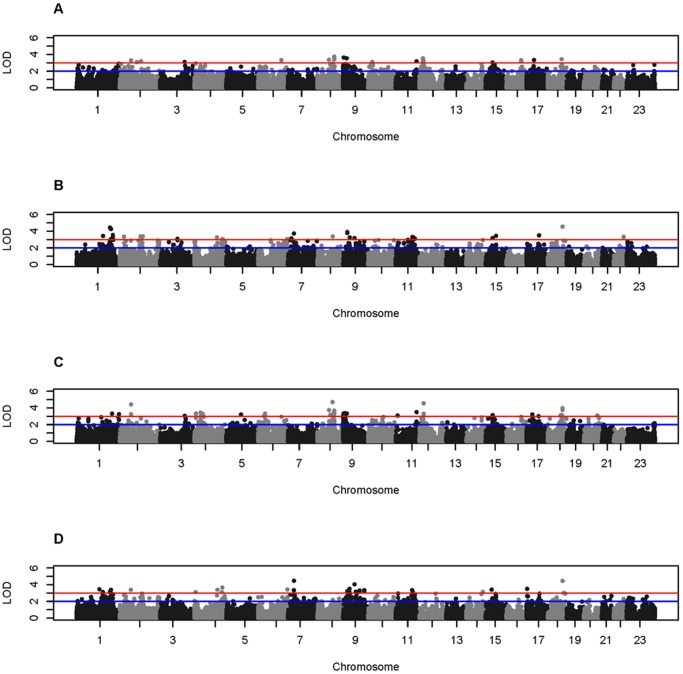
Whole genome two-point LOD scores obtained from stratified analysis. LOD score thresholds of 2 and 3 are indicated by the blue and red lines, respectively. HLOD scores for CTD-negative (A) and CTD-positive family subsets (B), and LOD scores under an exponential model for CTD-negative (C) and CTD-positive family subsets (D). LOD scores under a linear model are not shown as no two-point LOD scores exceeded 3. Negative two-point LOD scores are set to zero. Manhattan plots were created in R 2.15.0 using modified code obtained from “Getting Genetics Done” (http://gettinggeneticsdone.blogspot.com/).

**Figure 2:**
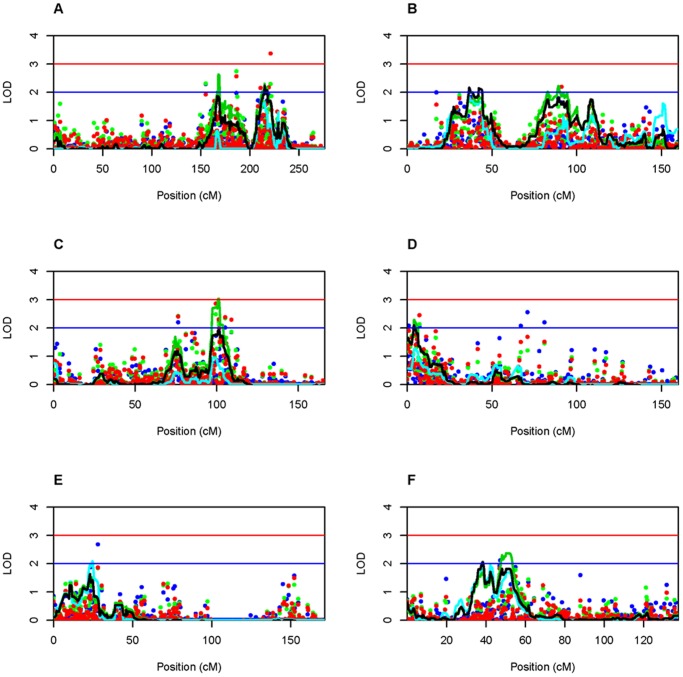
Two-point and multipoint LOD scores obtained from stratified analysis. Only chromosomes with a maximum multipoint LOD score >2 are shown. LOD score thresholds of 2 and 3 are indicated by the blue and red lines, respectively. Green points and lines represent LOD scores under a linear model, blue points and lines represent HLOD scores, and red points and black lines represent LOD scores under an exponential model. CTD-positive families: Chr1 (A), CTD-positive families: Chr9 (B), CTD-negative families: Chr8 (C), CTD-negative families: Chr9 (D), CTD-negative families: Chr12 (E), and CTD-negative families: Chr17 (F). Negative two-point and multipoint LOD scores are set to zero. Plots were created in R 2.15.0.

Families were stratified based on a family history of CTD related conditions and two-point and multipoint nonparametric and parametric whole genome linkage analyses were performed within the CTD-negative and CTD-positive group of families separately. Genome-wide results from the two-point analyses are shown in Figure 1 and the most significant two-point results are included in Table 2. As expected, different regions of the genome exhibit evidence for linkage depending on the subset of families examined. No two-point LOD scores under a linear model exceeded 3 within either family subset and were therefore not included in Figure 1 or Table 2. Maximum multipoint LOD scores exceeding 2 within either set of families are summarized in Figure 2 and Table 2. While no multipoint LOD scores exceeding 2 were previously obtained when all 66 families were analyzed collectively, multiple genomic regions now exhibit maximum LOD scores exceeding 2 and in one case exceeding 3 on chromosome 8. Notably, the most significant two-point LOD scores are found within the 1 LOD down supporting intervals for regions on chromosomes 8, 9, and 12 within the CTD-negative group of families and regions on chromosome 1 within the CTD-positive group of families (Table 2).

**Table 2:** Most significant two-point and multipoint LOD scores.^a^

Family description	Linkage model	Location (markers)^b^	Two-point LOD^c^	Emp p-value (CW/GW)^d^	Multipoint LOD^c^	Emp p-value (CW/GW)^d^
CTD-positive	Parametric: dominant	18q22.1 (rs17079623, rs574539)	***4.53***	**0.027**/0.150	0.71	0.787/1
		1q32.3 (rs2165993, rs3862952)	***4.42***	0.053/0.208	1.63	0.131/0.834
	NPL: exponential	7p15.3 (rs1476697, rs4719814)	***4.46***	**0.025**/0.553	0.57	0.601/1
		18q22.1 (rs17079623, rs2048329)	***4.44***	0.059/0.573	0.42	0.856/1
	NPL: linear	1q23.3-q24.2 (rs10494474)	0.87	1/1	**2.63**	**0.032**/0.184
		1q32.2-q41(rs3862952)	0.35	1/1	**2.3**	0.053/0.356
		9q21.31-q22.31 (rs10746837)	1.49	1/1	**2.22**	0.112/0.423
		9p22.3-p21.31 (rs2840790)	0.27	1/1	**2.15**	0.133/0.484
CTD-negative	Parametric: dominant	8q22.3 (rs12545537, rs544821)	***3.72***	0.156/0.871	0.01	1/1
		9p24.2 (rs2181829, rs7024139)	***3.62***	0.316/0.928	1.26	0.596/0.990
		12p13.31-p13.2 (rs6488255)	0.63	1/1	**2.09**	0.066/0.439
	NPL: exponential	8q22.1(rs1597301, rs6989464)	***4.69***	**0.031**/0.394	1.73	0.066/0.768
		12p13.2 (rs7312834, rs205534)	***4.54***	**0.014**/0.498	0.85	0.414/1
		17p12 (rs6502282)	0.08	1/1	**2.06**	**0.044**/0.491
	NPL: linear	8q21.3-q22.1 (rs7013599)	**2.21**	0.700/1	***3.04***	**0.008**/0.070
		17p12-q11.2 (rs7406339)	0.6	1/1	**2.37**	**0.027**/0.309
		9p24.3-p24.2 (rs1416621)	1.72	1/1	**2.29**	0.097/0.366

a The top two most significant two-point results within each model and family subset as well as any maximum multipoint LOD score exceeding 2 are included.

b When two markers are listed, the first corresponds to the marker used for the two-point result shown. The second corresponds to the closest marker included in the multipoint analysis.

c LOD scores exceeding 2 are bold and LOD scores exceeding 3 are bold and italicized. For the parametric model, HLOD scores are shown.

d Empirical p-values less than 0.05 are bold.

Abbreviations: CTD: connective tissue disorder, NPL: nonparametric linkage, LOD: logarithm of the odds, Emp: empirical, CW: chromosome-wide, GW: genome-wide, N/A: not applicable.

### Permutation Tests

In order to assess the relationship between the CTD stratification criteria and evidence for linkage, both genome-wide (GW) and chromosome-wide (CW) empirical p-values were obtained for both multipoint and two-point analyses under the three linkage models. Although no marker met GW significance, the peak marker for 8q21.3–q22.1 had a GW empirical p-value of 0.07 with a highly significant CW empirical p-value of 0.008. Additionally, several markers from the two-point and multipoint analyses had CW empirical p-values less than 0.05 as shown in Table 2. It is important to note that the empirical p-values derived from the permutation tests are approximate due to the fact that these families are of different sizes and structures.

### Candidate Gene Sequencing

Sanger sequencing was performed on all affected individuals from families with a positive LOD score for the linkage peak marker in the chromosome 8 or 12 linkage regions. The primary focus was on the most significant multipoint linkage peak found on chromosome 8 within the CTD-negative group of families (8q21.3–q22.1; Max LOD = 3.04, linear model). The 1 LOD down supporting interval contained 49 candidate RefSeq genes (Chr8∶91334498–98960813, GRCh37/hg19). Of those, one of particular interest was Growth differentiation factor 6 (GDF6) which is a member of the bone morphogenetic protein (BMP) sub-family and has been previously associated with a wide range of phenotypes including ocular, such as microphthalmia and coloboma, as well as skeletal, such as Klippel-Feil syndrome (KFS) which is characterized by fusion of any two of the seven cervical vertebrae [67], [68]. The candidate interval on chromosome 12p13.31–p13.2 (Max HLOD = 2.09, 1 LOD down interval: Chr12∶7794736–12721298, GRCh37/hg19) identified within a clinically similar subset of families (CTD-negative) also harbored a growth differentiation factor (GDF3), mutations in which have been previously associated with KFS [69]. As CMI and KFS may be allelic disorders, both GDF6 and GDF3 were selected for candidate gene sequencing in order to identify mutations and/or rare variants that increase susceptibility for disease.

**Figure 3:**
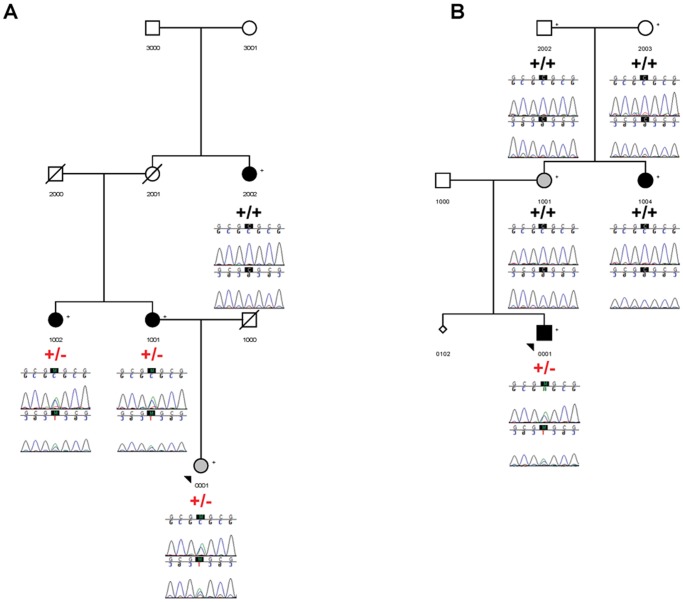
Segregation of the missense mutation, rs121909352 (A249E), in two CMI pedigrees. Family 9453 (A) and Family 9476 (B). Symbols shaded in black indicate a diagnosis of CMI with or without syringomyelia, small diamonds represent a miscarriage, and symbols shaded in grey indicate an uncertain diagnosis. 9453-0001 has been diagnosed with a suspected Chiari Malformation Type 0 and 9476–1001 has been diagnosed with tonsillar ectopia. “+/+” indicates homozygous for the reference allele; “+/−“ indicates heterozygous for the variant allele. Sequences were generated in both the forward and reverse direction and are shown below each sampled individual. Progeny 8 (Delray Beach, FL) was used to construct the pedigrees and Sequencher 5.0 (Ann Arbor, MI) was used to create the chromatograms.

In total, 22 SNPs, 2 insertions, and 1 deletion were found in GDF6 and 3 SNPs were found in GDF3 (Table S5). Of these, 6 were novel and 12 were rare (1000 Genomes European MAF <0.05) in GDF6 and 1 was rare in GDF3. In order to validate and establish segregation for a subset of these variants, 8 variants (7 in GDF6 and 1 in GDF3) were selected for follow-up sequencing (Table 3; see Methods under the candidate gene sequencing section for selection criteria). Within this subset of rare and novel variants, complete sharing across affected family members was observed with only two of the variants: 1) Novel SNP, g.406+2780C>T and 2) rs140757891. Reduced penetrance was observed for all variants of interest, except for rs121909352 although this is likely due to the fact that DNA samples were not available for all family members. Of particular interest is the missense variant, rs121909352 (A249E), which is a heterozygous mutation previously identified in KFS patients [67], [68] as well as patients with microphthalmia and coloboma [68], [70], [71]. Pedigrees showing segregation of this mutation with affection status are shown in Figure 3. Within family 9453, all individuals presenting with CMI and syringomyelia, except for individual 2002, were heterozygous for the mutation. Individual 2002 was found to have increased homozygosity as determined by an F inbreeding coefficient >4 standard deviations away from the mean and had been previously removed from the linkage analysis. In addition, one individual presenting with a suspected Chiari Malformation Type 0 (CM0) in family 9453 was heterozygous for the mutation; a detailed clinical description of this individual has been provided previously [72]. CM0 patients present with syringomyelia without tonsillar herniation that improves following posterior fossa decompression surgery. In family 9476, only one individual diagnosed with CMI and syringomyelia was heterozygous for the mutation (Figure 3); one additional individual with CMI and syringomyelia (1004) and one individual with tonsillar ectopia (1001) did not have the mutation.

**Table 3:** GDF6 and GDF3 selected sequence variants.^a^

Gene	Chr	Location^b^	Variant ID^c^	Alleles^d^	Variant Class	CMI/1KG MAF^e^	All Affsf,g	Reduced Pen^g^
GDF6	8	97154593	g.18328T>G	T/G	3′ UTR	0.005/NA	No (1/2)	Yes (1/2)
GDF6	8	97154813	rs112542818	C/T	3′ UTR	0.026/0.003	No (5/8)	Yes (2/4)
GDF6	8	97157223	rs148861809	C/G	Coding-syn	0.036/0.028	No (7/10)	Yes (3/8)
GDF6	8	97157413	rs121909352	G/T	Missense	0.016/0.003^h^	No (4/6)^i^	Unknown (0/3)
GDF6	8	97157811	g.15169-59T>A	T/A	Intronic	0.005/NA	No (1/2)	Yes (1/2)
GDF6	8	97169735	g.406+2780C>T	C/T	Intronic	0.010/NA	Yes (2/2)	Yes (1/2)
GDF6	8	97170374	rs140757891	C/T	Intronic	0.021/0.013	Yes (4/4)	Yes (2/10)
GDF3	12	7842587	rs2302516	C/G	Missense	0.047/0.024	No (7/11)	Yes (1/14)

a Only variants which were followed-up are shown here (See Methods section); Variants were validated by bidirectional sequencing and all sampled affected and unaffected individuals within each identified family were sequenced.

b Base pair positions based on human genome build GRCh37/hg19.

c The nomenclature used to describe novel variants was based on recommendations by the Human Genome Variation Society (den Dunnen and Antonarakis 2001). Nucleotide numbering was based on the GDF6 RefSeq genomic sequence, NG_008981.1, and intron-exon boundaries were defined based on the GDF6 mRNA sequence, NM_001001557.

d Alleles: Reference allele/Alternate allele.

e CMI MAF estimate based on all affected family members initially screened; 1KG MAF: Based on 1000 Genomes Integrated Phase 1 Release v3: European population.

f Is sharing observed across all affected individuals within each family?

g Numbers in parentheses: Numerator: number of sampled individuals carrying the variant, Denominator: total number of sampled individuals. Only affecteds were considered for “All affecteds” and only unaffecteds/uncertains were considered for “Reduced penetrance”.

h MAF estimate was not available from 1000 Genomes; MAF estimate based on the Exome sequencing project: European population.

i Individual suspected to have Chiari Malformation Type 0 is counted as “affected” for the purposes of this table.

In addition, the two intronic variants (Novel SNP, g.406+2780C>T and rs140757891) that are shared across all affected family members are located within potential regulatory regions (Table 3). The novel intronic SNP (g.406+2780C>T) is located within a predicted regulatory region for the protein, Suppressor of zeste 12 homolog (SUZ12), based on chromatin immunoprecipitation sequencing (ChIP-seq) data from the Encyclopedia of DNA Elements Consortium (ENCODE) (UCSC Genome browser: GRCh37/hg19 human assembly). The rare intronic SNP, rs140757891, is also located within a predicted regulatory region for SUZ12 as well as the GATA binding protein 2 (GATA2) based on ChIP-seq data from ENCODE (UCSC Genome browser: GRCh37/hg19 human assembly). In addition, rs140757891 is part of a CpG dinucleotide located within a predicted CpG island spanning 701 base pairs (UCSC Genome browser: GRCh37/hg19 human assembly). When the variant allele is present the guanine (G) becomes an adenine (A) (reverse strand). Segregation of these two intronic variants, along with two additional novel variants (g.18328T>G and g.15169-59T>A) found in one of these families are provided in Figure S2.

## Discussion

In order to gain a better understanding of the genetic architecture of CMI, we conducted a whole genome linkage screen using a collection of 66 nonsyndromic families with at least two sampled individuals presenting with CMI with or without syringomyelia. It was hypothesized that the limited evidence for linkage across all 66 families collectively was due to genetic heterogeneity and may be associated with the phenotypic variability observed. Based on the co-occurrence of CMI and CTDs, families were stratified by CTD related conditions in order to identify phenotypically and potentially genetically more homogeneous groups of families for linkage analysis. Stratified analyses identified multiple genomic regions showing increased evidence for linkage consistent with reduced genetic heterogeneity across families as a result of the CTD related stratification criteria. Furthermore, several plausible disease genes were identified as discussed in detail below.

Prior to describing our most significant results, it is important to relate our findings to the only other whole genome linkage screen conducted to date which implicated regions on chromosomes 9 and 15 [17]. We only identified suggestive evidence for linkage to the region on chromosome 9 within our CTD-positive group of families. Importantly, 12/66 of our total families and 7/34 CTD-positive families overlap with the families used in the initial screen conducted by Boyles and colleagues; therefore, these results do not provide independent replication for this region. Lack of replication for chromosome 15 could be due to the use of: 1) different genotyping chips (Illumina Human610-Quad BeadChips versus Affymetrix 10K SNP Chip) and marker quality control procedures, 2) different linkage software packages (Merlin versus Allegro; e.g. different with respect to an error detection option and accounting for inter-marker LD) and genetic models (penetrance function and S scoring function), 3) additional families which are likely genetically heterogeneous, and/or 4) different analytical approaches (stratified analyses). While the original finding could be a false positive, it is equally possible that as additional families are collected and other approaches to reduce genetic heterogeneity are applied to the data this region may present again as a promising candidate genomic interval warranting follow-up.

While we presented linkage results within the subsets of both CTD-positive and CTD-negative families, the focus of the current paper has been on the CTD-negative families as these are thought to represent more “classical” CMI due to cranial constriction and also resulted in the identification of the only genomic region with a maximum LOD score exceeding 3. The most significant of our findings implicated the growth differentiation factors, GDF6 and GDF3, both of which had been previously implicated in KFS [67], [68], [69] which is characterized by cervical vertebral fusion and may be associated with a wide range of conditions including renal abnormalities, cardiovascular abnormalities, orthopedic anomalies, pulmonary problems, deafness, and synkinesia [73]. Interestingly, roughly 3–5% of CMI patients are diagnosed with KFS [23], [45], suggesting a shared genetic etiology between these disorders. Further, it has been proposed that KFS and CMI should be classified as post-otic neural crest syndromes, thus sharing a common cellular etiology [74]. Although the exact relationship between these disorders is unknown, one possibility is that CMI and KFS may be allelic disorders. In order to investigate this possibility, GDF3 and GDF6 were sequenced in a collection of CMI patients from our linkage families. While GDF3 still presents as an intriguing biological candidate and additional sequencing of potential regulatory elements may yield putative disease variants, no variants of obvious significance were identified in this study. However, several interesting variants were identified in GDF6. A previously identified KFS mutation, A249E (rs121909352), was found in two of our CMI families. The functional effect of this mutation has been determined previously *in-vitro*. Asai-Coakwell and colleagues evaluated changes to bone morphogenetic protein (BMP) signaling by co-transfecting an expression construct with the A249E mutation and a Sex determining region Y-box 9 (SOX-9)-responsive reporter gene into primary limb mesenchymal cells and assessed SOX-9 reporter activity [68]. Reduced activation of the reporter was observed (p<0.034), suggesting altered chondrogenic potential [68]. In addition, a 23% reduction in secreted mature GDF6 protein expression was observed for the mutant as determined by Western blot analysis [68].

Although there is evidence for a functional effect, the expression of A249E is complex with previous evidence of pleiotropy (ocular versus skeletal phenotypes), variable expressivity (e.g. coloboma versus microphthalmia), and reduced penetrance [68]. Consistent with these reports, we also observe variable expressivity within our CMI families (CMI with syringomyelia versus CM0). In fact, the identification of A249E in both CMI and a suspected CM0 individual within the same family (9453) further supports the hypothesis that these disorders share an underlying genetic basis and represent part of a continuum of Chiari phenotypes [72], [75], [76], [77], [78]. Although A249E is not necessary to cause disease in either of these families, it still likely contributes to disease presentation together with additional genetic and potentially environmental factors.

Additional variants of interest from our study include two intronic GDF6 variants, rs140757891 and a novel SNP, g.406+2780C>T. ChIP-seq data from a small number of cell lines indicate that both variants are located within predicted targets of SUZ12, a polycomb protein involved in epigenetic silencing of developmental genes. Interestingly, haploinsufficient SUZ12 mice exhibit cerebellar herniation, as well as spina bifida, an enlarged brainstem, and occipital cortical changes [79]. Although these clinical features appear to be due to an enlarged tectum and only demonstrate partial clinical similarity with CMI, Miro and colleagues suggest that an additional link between SUZ12 and central nervous system disorders may come from neurofibromatosis 1 (NF1), a disorder characterized by the development of neurofibromas and the presence of café-au-lait spots [79]. SUZ12 and NF1 are located within 560 kb of each other on chromosome 17 and while most NF1 patients have point mutations in NF1 some harbor larger genomic deletions that encompass NF1 as well as other genes, including SUZ12 resulting in a more severe clinical presentation [79]. Roughly 5% of CMI patients present with NF1 [45] and it has been previously suggested that these two disorders may share an underlying genetic basis [58]. Remarkably, within the same group of families that showed increased evidence for linkage to the region containing GDF6 (CTD-negative) we also observed suggestive evidence for linkage to 17p12–q11.2 (Max LOD = 2.37, CW emp p-val = 0.03) which contains both SUZ12 and NF1 providing further support for a potential role in disease development.

While encouraged by our findings, we acknowledge several limitations of this study. First, because we enforced strict eligibility criteria (exclusion of syndromic cases) and required families to have multiple affected individuals, the total number of families eligible for the study was low and likely contributed to reduced power. However, despite the relatively small sample size, the number of families examined was almost three times as large as the collection of families used in the only other whole genome linkage screen published to date [17]. Second, MRIs were not available for all study participants thus misclassification of affection status cannot be ruled out. Importantly, none of our analyses used phenotype information from “unaffected” family members (i.e. affecteds-only analysis), thus the greatest impact of potential misclassification would be if individuals were incorrectly classified as affected. Furthermore, clinical information used for the stratified analysis was mostly ascertained through a general medical interview upon enrollment in the study; therefore, misclassification of families as CTD-positive or CTD-negative is possible. Nevertheless, our data suggest that the increased evidence for linkage observed for the stratified analysis based on CTD related conditions is non-random (e.g. 8q21.3–q22.1: GW emp p-val = 0.07, CW emp p-val = 0.008). This observation would seem unlikely if a high degree of misclassification existed.

Future work will include functional follow-up of variants of interest as well as sequencing GDF3 and GDF6 in a larger cohort of sporadic and familial CMI cases. Furthermore, the distant regulatory elements previously identified for GDF6 [80], [81], [82] represent excellent candidate regions for future *de novo* variant detection. Other candidate genes, such as low density lipoprotein receptor-related protein 6 (LRP6) present within the chromosome 12 candidate interval could also be investigated as LRP6, when specifically deleted from early mesenchyme, causes a slight delay in mouse skull ossification [83]. In addition, rather than simply taking a candidate gene approach, targeted capture and next generation sequencing of candidate genomic intervals defined by linkage analysis or whole genome sequencing would be an obvious next step to comprehensively follow-up these findings. Finally, taking a more quantitative approach to disease, for example by focusing on cranial base morphometrics, may yield greater insight into the genetic etiology due to increased statistical power and reduced misclassification rates among individuals.

### Conclusion

The current study demonstrates the utility of using clinical stratification to reduce genetic heterogeneity in CMI by identifying genomic regions showing increased evidence for linkage with maximum LOD scores exceeding 2 and even 3, as well as having implicated credible candidate genes in CMI susceptibility. Although further work is necessary to confirm the involvement of these genes and individual sequence variants in the development of CMI, this work makes several important contributions to the field of CMI research: 1) We conducted the largest whole genome linkage screen to date providing multiple candidate intervals for future investigation and replication, 2) Our results suggest a relationship between CTD related conditions and genetic etiology which is consistent with the hypothesis that CMI with CTDs versus CMI without CTDs occur through different mechanisms (“cranial settling” versus “cranial constriction”), 3) Multiple biological candidates were implicated from the analysis, including the only two GDFs currently known to be associated with KFS suggesting a shared genetic etiology between CMI and KFS. This is consistent with the fact that KFS is known to co-occur with CMI and share a common cellular etiology, 4) Identified a known KFS missense mutation in two of our families that is not necessary for disease but likely contributes to the phenotype due to its rare frequency in the general population, known functional effect *in vitro*, and the fact that it has been identified in multiple skeletal and ocular disease cohorts, and 5) Identified two potential regulatory variants (one novel, one rare) shared across all affected individuals in the families they were identified in and located within predicted regulatory regions for SUZ12 which itself is an excellent candidate gene for CMI. Further investigation of GDF3 and GDF6, other plausible biological candidates such as SUZ12, NF1, and LRP6, as well as the genetic relationship between CMI and KFS is warranted.

## Supporting Information

Figure S1 **Multidimensional scaling (MDS) analysis.** This was performed using a pruned marker dataset and only one representative individual from each family. The red and black colors correspond to different clusters (PLINK's pairwise population concordance test: –ppc 1e-4). Individuals shown in red represent families that are self-reported Caucasian, Hispanic. MDS plot was created in R 2.15.0.
(TIFF)

Figure S2 **Segregation of select variants in three CMI pedigrees.** Family 9772 (A), Family 9496 (B), and Family 9432 (C). Alleles “A” and “a” represent a novel SNP at Chr8∶97154593, Alleles “B” and “b” represent a novel SNP at Chr8∶97157811, alleles “C” and “c” represent RS140757891, and alleles “D” and “d” represent a novel SNP at Chr8∶97169735. Individual 108 from family 9496 has previously had brain surgery and a shunt; no additional information is known. Symbols shaded in black indicate a diagnosis of CMI with or without syringomyelia and small diamonds represent miscarriages. Lower case letters shown in red indicate the variant allele. Genotype calls are based on bidirectional sequencing. Progeny 8 (Delray Beach, FL) was used to construct the pedigrees.
(TIFF)

Table S1 **PCR primer sequences and conditions.**
(DOC)

Table S2 **PCR primer kits and thermocycler conditions.**
(DOC)

Table S3 **Quality control of sample data.**
(DOC)

Table S4 **Most significant two-point and multipoint LOD scores.**
(DOC)

Table S5 **GDF6 and GDF3 identified sequence variants.**
(DOC)
