# Supplementary figures and images for: Stratified Whole Genome Linkage Analysis of Chiari Type I Malformation Implicates Known Klippel-Feil Syndrome Genes as Putative Disease Candidates
Source: PLoS One. 2013 Apr 19;8(4):e61521. doi: 10.1371/journal.pone.0061521 (PMC3631233; doi:10.1371/journal.pone.0061521)

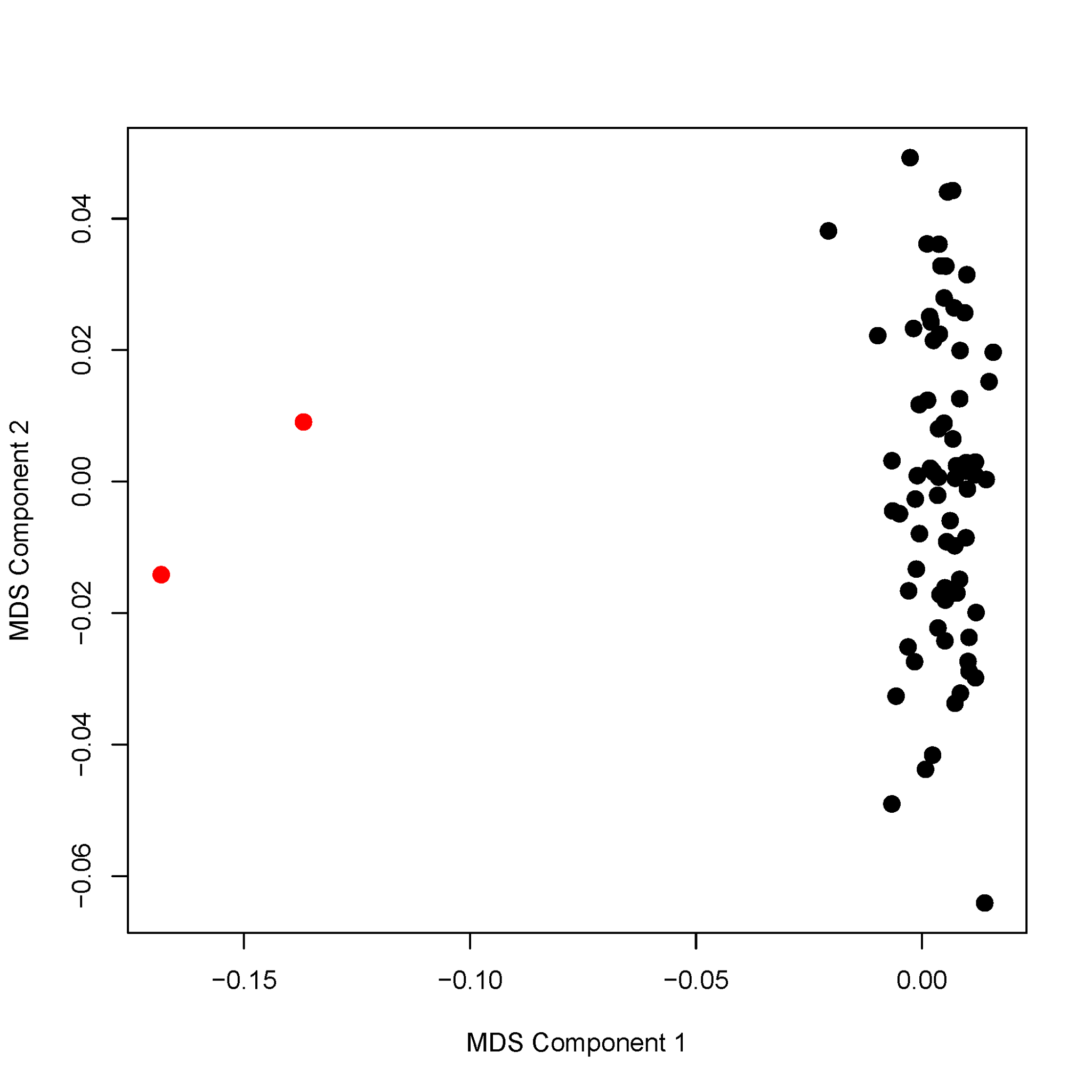

Supplement: Figure S1 — Multidimensional scaling (MDS) analysis. This was performed using a pruned marker dataset and only one representative individual from each family. The red and black colors correspond to different clusters (PLINK's pairwise population concordance test: –ppc 1e-4). Individuals shown in red represent families that are self-reported Caucasian, Hispanic. MDS plot was created in R 2.15.0. (TIFF) [file pone.0061521.s001.tiff]

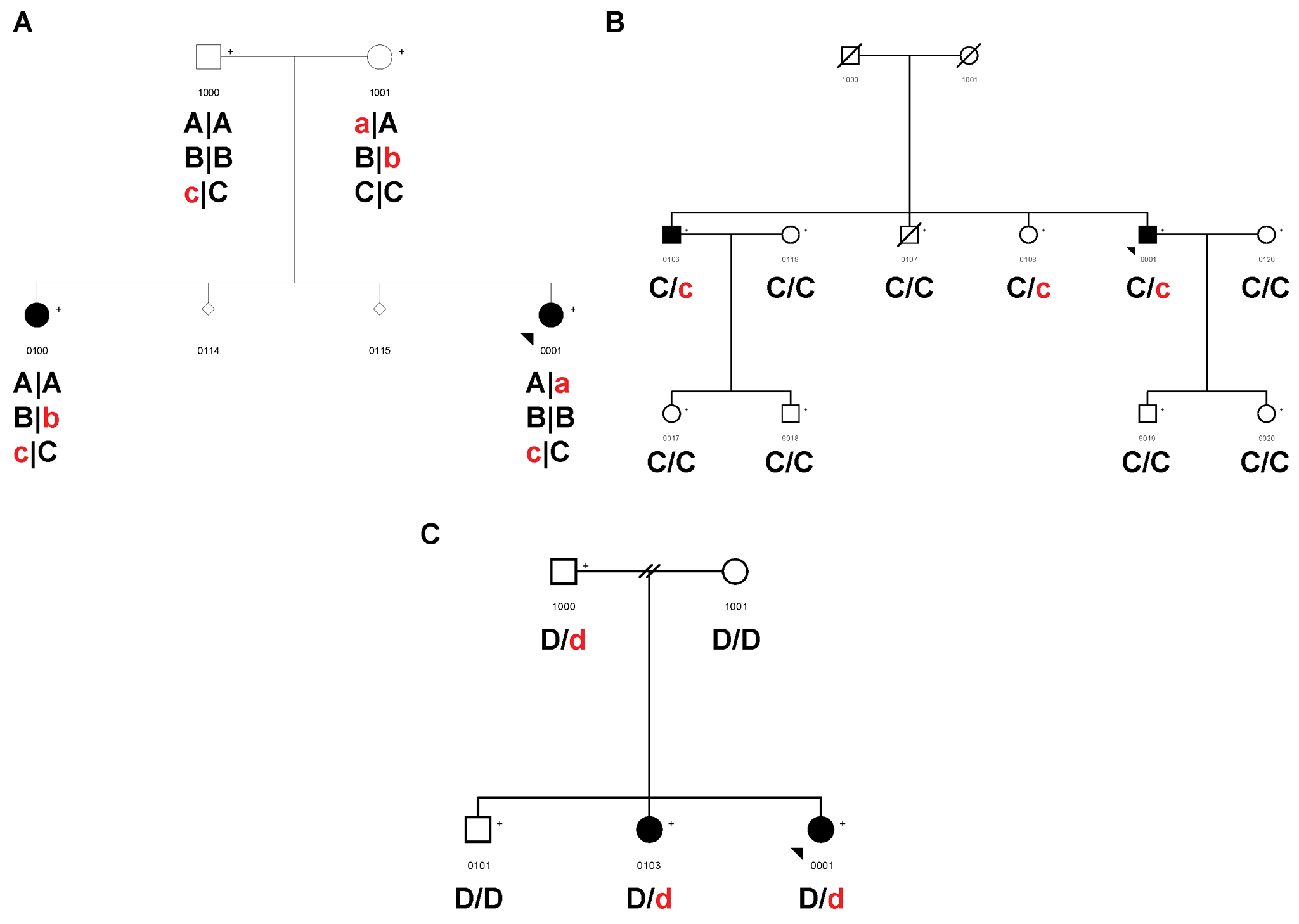

Supplement: Figure S2 — Segregation of select variants in three CMI pedigrees. Family 9772 (A), Family 9496 (B), and Family 9432 (C). Alleles “A” and “a” represent a novel SNP at Chr8∶97154593, Alleles “B” and “b” represent a novel SNP at Chr8∶97157811, alleles “C” and “c” represent RS140757891, and alleles “D” and “d” represent a novel SNP at Chr8∶97169735. Individual 108 from family 9496 has previously had brain surgery and a shunt; no additional information is known. Symbols shaded in black indicate a diagnosis of CMI with or without syringomyelia and small diamonds represent miscarriages. Lower case letters shown in red indicate the variant allele. Genotype calls are based on bidirectional sequencing. Progeny 8 (Delray Beach, FL) was used to construct the pedigrees. (TIFF) [file pone.0061521.s002.tiff]
