# Supplementary material for: Stratified Whole Genome Linkage Analysis of Chiari Type I Malformation Implicates Known Klippel-Feil Syndrome Genes as Putative Disease Candidates
Source: PLoS One. 2013 Apr 19;8(4):e61521. doi: 10.1371/journal.pone.0061521 (PMC3631233; doi:10.1371/journal.pone.0061521)
Supplement: Table S1 — PCR primer sequences and conditions. (DOC) [file pone.0061521.s003.doc]

**Table S1** PCR primer sequences and conditions

| **Gene** | **Amplicon** | **Coordinates^a^** | **Forward primer** | **Reverse primer** |
| --- | --- | --- | --- | --- |
| GDF6 | Exon1-1 | chr8:97172588-97173187 | CCCTCTCCCCCACACCTC | CAGCTTCTCAGCGATGGAGT |
| GDF6 | Exon1-2 | chr8:97172349-97172848 | CAGGCTTCCATCTCATCCTC | GCCACATTCAGAAACTTACTCG |
| GDF6 | Intron1-1 | chr8:97169979-97170507 | ACGAGACACCGTTTGATGTG | TAGAGGAGGGATTGCATGTG |
| GDF6 | Intron1-2 | chr8:97169568-97170159 | ATGCCTGTTCTCCCACTCTC | CCCAGCTTGATAAAGGGAAAG |
| GDF6 | Intron2 | chr8:97167727-97168244 | CTTTTTGCAGGACCTTCCAG | GCAGGACCATTCTGGGAATA |
| GDF6 | Intron3-1 | chr8:97159214-97159794 | GGCTGCCTTCCCATAAAATA | AACTGTTCTGTGCAGGTGGA |
| GDF6 | Intron3-2 | chr8:97158975-97159469 | TCCTCCCACTCCCAATCTAA | ATCTCAGGTTGGGGCATGT |
| GDF6 | Exon2-1 | chr8:97157502-97158060 | GTGGTTACGAAGCCTTTTGG | CTGCCACACGTCGAAGACT |
| GDF6 | Exon2-2 | chr8:97157009-97157600 | CAGCTCTTCCCTTGCCTTTC | CGCAGTGATAGGCCTCGTA |
| GDF6 | Exon2-3 | chr8:97156568-97157120 | GGCAAGAAGTCCAGGCTACG | GGCAAGGTGTGAAAATCCAT |
| GDF6 | Exon2-4 | chr8:97156319-97156954 | TCATCCAGACGCTGATGAAC | CGTCAACGGTGATTCTTCCT |
| GDF6 | Exon2-5 | chr8:97155948-97156445 | TGTCTTACAGGCTTTGATAGAAGG | CAAGGGGTTCCATTTGGACT |
| GDF6 | Exon2-6 | chr8:97155544-97156041 | CCAGGTCTCTGCCTTCATTG | AAGAGAGGGGAGGGAACTCA |
| GDF6 | Exon2-7 | chr8:97155191-97155688 | CATCAGGAGGGAAGATGGTG | AGTCAAAGATGAGGAGGAAGC |
| GDF6 | Exon2-8 | chr8:97154891-97155371 | GAAGGAGACAGGGGTGGAAT | GCTGTCCATTTCCTCTTTGC |
| GDF6 | Exon2-9 | chr8:97154598-97155066 | AAAGAAAGGGTGGGGATGAT | TTGCATATCATCCAGGAAAGG |
| GDF6 | Exon2-10 | chr8:97154342-97154910 | GCAAAGAGGAAATGGACAGC | GGTGACAAGGATTTGGCTTT |
| GDF3 | Exon1 | chr12:7847952-7848454 | TGGCCTTTGAGGAGCTG | AATACCAGCACAAGGCCATC |
| GDF3 | Exon2-1 | chr12:7842771-7843420 | AGGGCTTGTGTCTTTCCCTG | ACGGTGGCAGAGGTTCTTAC |
| GDF3 | Exon2-2 | chr12:7842270-7842915 | TGAAGACACCTGTGCCAGAC | AACTATGATTATTAGGGCTCCAGG |

^a^Base pair positions based on the GRCh37/hg19 human genome assembly
