# Supplementary material for: Stratified Whole Genome Linkage Analysis of Chiari Type I Malformation Implicates Known Klippel-Feil Syndrome Genes as Putative Disease Candidates
Source: PLoS One. 2013 Apr 19;8(4):e61521. doi: 10.1371/journal.pone.0061521 (PMC3631233; doi:10.1371/journal.pone.0061521)
Supplement: Table S2 — PCR primer kits and thermocycler conditions. (DOC) [file pone.0061521.s004.doc]

**Table S2** PCR primer kits and thermocycler conditions

| **Gene** | **Amplicon** | **PCR kit** | **Thermocycler conditions** |
| --- | --- | --- | --- |
| GDF6 | Exon1-1 | Invitrogen Accuprime™ GC Rich DNA Polymerase kit | 95°C for 3 min; 94°C for 30 sec, 66°C for 30 sec (-1°C each cycle at 50% ramp speed), 72°C for 1:45 min (13 cycles); 94°C for 30 sec, 52°C for 45 sec, 72°C for 1:45 min (25 cycles); 72°C for 10 min |
| GDF6 | Exon1-2 | Qiagen HotStarTaq® Plus with Q solution | 95°C for 5 min; 94°C for 30 sec, 66°C for 30 sec (-1°C each cycle), 72°C for 1:45 min (13 cycles); 94°C for 10 sec, 52°C for 45 sec, 72°C for 1:45 min (25 cycles); 72°C for 8 min |
| GDF6 | Intron1-1 | Qiagen HotStarTaq® Plus with Q solution | 95°C for 5 min; 94°C for 30 sec, 66°C for 30 sec (-1°C each cycle), 72°C for 1:45 min (13 cycles); 94°C for 10 sec, 52°C for 45 sec, 72°C for 1:45 min (25 cycles); 72°C for 8 min |
| GDF6 | Intron1-2 | Qiagen HotStarTaq® Plus with Q solution | 95°C for 5 min; 94°C for 30 sec, 66°C for 30 sec (-1°C each cycle), 72°C for 1:45 min (13 cycles); 94°C for 10 sec, 52°C for 45 sec, 72°C for 1:45 min (25 cycles); 72°C for 8 min |
| GDF6 | Intron2 | Qiagen HotStarTaq® Plus with Q solution | 95°C for 5 min; 94°C for 30 sec, 66°C for 30 sec (-1°C each cycle), 72°C for 1:45 min (13 cycles); 94°C for 10 sec, 52°C for 45 sec, 72°C for 1:45 min (25 cycles); 72°C for 8 min |
| GDF6 | Intron3-1 | Qiagen HotStarTaq® Plus with Q solution | 95°C for 5 min; 94°C for 30 sec, 66°C for 30 sec (-1°C each cycle), 72°C for 1:45 min (13 cycles); 94°C for 10 sec, 52°C for 45 sec, 72°C for 1:45 min (25 cycles); 72°C for 8 min |
| GDF6 | Intron3-2 | Qiagen HotStarTaq® Plus with Q solution | 95°C for 5 min; 94°C for 30 sec, 66°C for 30 sec (-1°C each cycle), 72°C for 1:45 min (13 cycles); 94°C for 10 sec, 52°C for 45 sec, 72°C for 1:45 min (25 cycles); 72°C for 8 min |
| GDF6 | Exon2-1 | Qiagen HotStarTaq® Plus with Q solution | 95°C for 5 min; 94°C for 30 sec, 66°C for 30 sec (-1°C each cycle), 72°C for 1:45 min (13 cycles); 94°C for 10 sec, 52°C for 45 sec, 72°C for 1:45 min (25 cycles); 72°C for 8 min |
| GDF6 | Exon2-2 | Invitrogen Accuprime™ GC Rich DNA Polymerase kit | 95°C for 3 min; 94°C for 30 sec, 56°C for 30 sec, 72°C for 1 min (30 cycles); 72°C for 10 min |
| GDF6 | Exon2-3 | Qiagen HotStarTaq® Plus with Q solution | 95°C for 5 min; 94°C for 30 sec, 66°C for 30 sec (-1°C each cycle), 72°C for 1:45 min (13 cycles); 94°C for 10 sec, 52°C for 45 sec, 72°C for 1:45 min (25 cycles); 72°C for 8 min |
| GDF6 | Exon2-4 | Qiagen HotStarTaq® Plus with Q solution and 2.5mM MgCl_2_ | 95°C for 5 min; 94°C for 30 sec, 66°C for 30 sec (-1°C each cycle), 72°C for 1:45 min (13 cycles); 94°C for 10 sec, 52°C for 45 sec, 72°C for 1:45 min (25 cycles); 72°C for 8 min |
| GDF6 | Exon2-5 | Qiagen HotStarTaq® Plus with Q solution | 95°C for 5 min; 94°C for 30 sec, 66°C for 30 sec (-1°C each cycle), 72°C for 1:45 min (13 cycles); 94°C for 10 sec, 52°C for 45 sec, 72°C for 1:45 min (25 cycles); 72°C for 8 min |
| GDF6 | Exon2-6 | Qiagen HotStarTaq® Plus with Q solution | 95°C for 5 min; 94°C for 30 sec, 66°C for 30 sec (-1°C each cycle), 72°C for 1:45 min (13 cycles); 94°C for 10 sec, 52°C for 45 sec, 72°C for 1:45 min (25 cycles); 72°C for 8 min |
| GDF6 | Exon2-7 | Qiagen HotStarTaq® Plus with Q solution | 95°C for 5 min; 94°C for 30 sec, 66°C for 30 sec (-1°C each cycle), 72°C for 1:45 min (13 cycles); 94°C for 10 sec, 52°C for 45 sec, 72°C for 1:45 min (25 cycles); 72°C for 8 min |
| GDF6 | Exon2-8 | Qiagen HotStarTaq® Plus with Q solution | 95°C for 5 min; 94°C for 30 sec, 66°C for 30 sec (-1°C each cycle), 72°C for 1:45 min (13 cycles); 94°C for 10 sec, 52°C for 45 sec, 72°C for 1:45 min (25 cycles); 72°C for 8 min |
| GDF6 | Exon2-9 | Qiagen HotStarTaq® Plus without Q solution | 95°C for 5 min; 94°C for 30 sec, 66°C for 30 sec (-1°C each cycle), 72°C for 1:45 min (13 cycles); 94°C for 10 sec, 52°C for 45 sec, 72°C for 1:45 min (25 cycles); 72°C for 8 min |
| GDF6 | Exon2-10 | Qiagen HotStarTaq® Plus without Q solution | 95°C for 5 min; 94°C for 30 sec, 66°C for 30 sec (-1°C each cycle), 72°C for 1:45 min (13 cycles); 94°C for 10 sec, 52°C for 45 sec, 72°C for 1:45 min (25 cycles); 72°C for 8 min |
| GDF3 | Exon1 | Qiagen HotStarTaq® Plus without Q solution | 95°C for 5 min; 94°C for 30 sec, 66°C for 30 sec (-1°C each cycle), 72°C for 1:45 min (13 cycles); 94°C for 10 sec, 52°C for 45 sec, 72°C for 1:45 min (25 cycles); 72°C for 8 min |
| GDF3 | Exon2-1 | Qiagen HotStarTaq® Plus without Q solution | 95°C for 5 min; 94°C for 30 sec, 66°C for 30 sec (-1°C each cycle), 72°C for 1:45 min (13 cycles); 94°C for 10 sec, 52°C for 45 sec, 72°C for 1:45 min (25 cycles); 72°C for 8 min |
| GDF3 | Exon2-2 | Qiagen HotStarTaq® Plus without Q solution | 95°C for 5 min; 94°C for 30 sec, 66°C for 30 sec (-1°C each cycle), 72°C for 1:45 min (13 cycles); 94°C for 10 sec, 52°C for 45 sec, 72°C for 1:45 min (25 cycles); 72°C for 8 min |
