# Supplementary material for: Stratified Whole Genome Linkage Analysis of Chiari Type I Malformation Implicates Known Klippel-Feil Syndrome Genes as Putative Disease Candidates
Source: PLoS One. 2013 Apr 19;8(4):e61521. doi: 10.1371/journal.pone.0061521 (PMC3631233; doi:10.1371/journal.pone.0061521)
Supplement: Table S3 — Quality control of sample data. (DOC) [file pone.0061521.s005.doc]

**Table S3 Quality control of sample data**

| **Description** | **Individuals** | | **Families** | |
| --- | --- | --- | --- | --- |
|  | **N_Excl** | **N_Total** | **N_Excl** | **N_Total** |
| Starting number |  | 422 |  | 73 |
| Exclusion criteria |  |  |  |  |
| Call rate < 99% | 0 | 422 | 0 | 73 |
| Per-family Mendelian errors > 2% | 0 | 422 | 0 | 73 |
| Sex discrepancy | 0 | 422 | 0 | 73 |
| Inbreeding coefficient > 4 SD from the mean^a^ | 3 | 419 | 0 | 73 |
| MDS outlier detection^b^ | 5 | 414 | 2 | 71 |
| Monozygotic twin pair (excluded 1/pair) | 4 | 410 | 1 | 70 |
| Families trimmed for linkage | 8 | 402 | 0 | 70 |
| No longer meets inclusion criteria^c^ | 35 | 367 | 4 | 66 |

^a^F inbreeding coefficient: Mean= -0.003; SD= 0.01

^b^Both families are self-reported Hispanic

^c^Family history of Ehlers-Danlos syndrome or singleton family

Abbreviations: N_Excl: number of individuals/families excluded, N_Total: total number of families remaining, SD: standard deviation
