# Supplementary material for: Stratified Whole Genome Linkage Analysis of Chiari Type I Malformation Implicates Known Klippel-Feil Syndrome Genes as Putative Disease Candidates
Source: PLoS One. 2013 Apr 19;8(4):e61521. doi: 10.1371/journal.pone.0061521 (PMC3631233; doi:10.1371/journal.pone.0061521)
Supplement: Table S4 — Most significant two-point and multipoint LOD scores. (DOC) [file pone.0061521.s006.doc]

**Table S4** Most significant two-point and multipoint LOD scores^a^

| **Family description** | **Linkage model** | **Location (markers)^b^** | **Two-point LOD^c^** | **Emp p-value (CW/GW)^d^** | **Multipoint LOD^c^** | **Emp p-value (CW/GW)^d^** |
| --- | --- | --- | --- | --- | --- | --- |
| All families | Parametric: dominant | 18q22.1 (rs17079623, rs2048329) | ***5.04*** | N/A | 1.24 | N/A |
|  |  | 8q22.1 (rs2513796, rs2446871) | ***4.62*** | N/A | 0.96 | N/A |
|  |  | 4q34.3 (rs17068194, rs1380000) | ***4.14*** | N/A | 0.08 | N/A |
|  |  | 1q32.2 (rs10863712, rs4844686) | ***3.94*** | N/A | 0.64 | N/A |
|  | NPL: exponential | 6p25.3 (rs11754896, rs11753072) | ***4.53*** | N/A | 0.02 | N/A |
|  |  | 1q32.2 (rs10863712, rs4844686) | ***4.21*** | N/A | 0.68 | N/A |
|  |  | 4q34.3 (rs17068194, rs1380000) | ***4.19*** | N/A | 0.28 | N/A |
|  |  | 9p24.1 (rs7875477, rs17630650) | ***4.08*** | N/A | 0.38 | N/A |
|  | NPL: linear | 1q32.2 (rs10863712, rs4844686) | ***3.57*** | N/A | 0.75 | N/A |
|  |  | 17p13.3 (rs7222425, rs9303183) | ***3.22*** | N/A | 0.61 | N/A |
|  |  | 8q22.1 (rs13268209, rs3104916) | ***3.18*** | N/A | 1.16 | N/A |
|  |  | 7p15.1 (rs160346, rs310338) | ***3.14*** | N/A | 0.49 | N/A |
|  |  |  |  |  |  |  |
| CTD-positive | Parametric: dominant | 18q22.1 (rs17079623, rs574539) | ***4.53*** | **0.027** / 0.150 | 0.71 | 0.787 / 1 |
|  |  | 1q32.3 (rs2165993, rs3862952) | ***4.42*** | 0.053 / 0.208 | 1.63 | 0.131 / 0.834 |
|  |  | 1q41 (rs1833036) | ***4.25*** | 0.081 / 0.335 | 0.86 | 0.628 / 1 |
|  |  | 9p23 (rs4740551, rs933034) | ***3.89*** | 0.157 / 0.704 | 1.34 | 0.547 / 0.971 |
|  | NPL: exponential | 7p15.3 (rs1476697, rs4719814) | ***4.46*** | **0.025** / 0.553 | 0.57 | 0.601 / 1 |
|  |  | 18q22.1 (rs17079623, rs2048329) | ***4.44*** | 0.059 / 0.573 | 0.42 | 0.856 / 1 |
|  |  | 9q21.2 (rs12379174, rs1343396) | ***4.03*** | 0.275 / 0.866 | 0.44 | 0.983 / 1 |
|  |  | 4q34.3 (rs6836317, rs1349923) | ***3.65*** | 0.221 / 0.979 | 1.03 | 0.181 / 1 |
|  | NPL: linear | 1q23.3-q24.2 (rs10494474) | 0.87 | 1 / 1 | **2.63** | **0.032** / 0.184 |
|  |  | 1q32.2-q41(rs3862952) | 0.35 | 1 / 1 | **2.30** | 0.053 / 0.356 |
|  |  | 9q21.31-q22.31 (rs10746837) | 1.49 | 1 / 1 | **2.22** | 0.112 / 0.423 |
|  |  | 9p22.3-p21.31 (rs2840790) | 0.27 | 1 / 1 | **2.15** | 0.133 / 0.484 |
|  |  |  |  |  |  |  |
| CTD-negative | Parametric: dominant | 8q22.3 (rs12545537, rs544821) | ***3.72*** | 0.156 / 0.871 | 0.01 | 1 / 1 |
|  |  | 9p24.2 (rs2181829, rs7024139) | ***3.62*** | 0.316 / 0.928 | 1.26 | 0.596 / 0.990 |
|  |  | 9p23 (rs833417,rs833413) | ***3.53*** | 0.383 / 0.964 | 0.13 | 1 / 1 |
|  |  | 12p13.31 (rs7963223, rs2377419) | ***3.52*** | 0.063 / 0.966 | 1.92 | 0.093 / 0.560 |
|  |  | 12p13.31-p13.2 (rs6488255) | 0.63 | 1 / 1 | **2.09** | 0.066 / 0.439 |
|  | NPL: exponential | 8q22.1(rs1597301, rs6989464) | ***4.69*** | **0.031** / 0.394 | 1.73 | 0.066 / 0.768 |
|  |  | 12p13.2 (rs7312834, rs205534) | ***4.54*** | **0.014** / 0.498 | 0.85 | 0.414 / 1 |
|  |  | 2p16.3 (rs3792246, rs6545061) | ***4.41*** | 0.074 / 0.591 | 0 | 1 / 1 |
|  |  | 18q22.1 (rs11151413, rs1518022) | ***3.97*** | 0.145 / 0.899 | 1.06 | 0.487 / 0.999 |
|  |  | 17p12 (rs6502282) | 0.08 | 1 / 1 | **2.06** | **0.044** / 0.491 |
|  | NPL: linear | 8q21.3-q22.1 (rs7013599) | **2.21** | 0.700 / 1 | ***3.04*** | **0.008** / 0.070 |
|  |  | 17p12-q11.2 (rs7406339) | 0.60 | 1 / 1 | **2.37** | **0.027** / 0.309 |
|  |  | 9p24.3-p24.2 (rs1416621) | 1.72 | 1 / 1 | **2.29** | 0.097 / 0.366 |

^a^The top four most significant two-point results within each model and family subset as well as any maximum multipoint LOD score exceeding 2 are included.

^b^When two markers are listed, the first corresponds to the marker used for the two-point result shown. The second corresponds to the closest marker included in the multipoint analysis.

^c^LOD scores exceeding 2 are bold and LOD scores exceeding 3 are bold and italicized. For the parametric model, HLOD scores are shown.

^d^Empirical p-values less than 0.05 are bold.

Abbreviations: CTD: connective tissue disorder, NPL: nonparametric linkage, LOD: logarithm of the odds, Emp: empirical, CW: chromosome-wide, GW: genome-wide, N/A: not applicable

|  |  |
| --- | --- |
|  | |
