# Supplementary material for: Stratified Whole Genome Linkage Analysis of Chiari Type I Malformation Implicates Known Klippel-Feil Syndrome Genes as Putative Disease Candidates
Source: PLoS One. 2013 Apr 19;8(4):e61521. doi: 10.1371/journal.pone.0061521 (PMC3631233; doi:10.1371/journal.pone.0061521)
Supplement: Table S5 — GDF6 and GDF3 identified sequence variants. (DOC) [file pone.0061521.s007.doc]

**Table S5** GDF6 and GDF3 identified sequence variants

| **Gene** | **Chr** | **Location^a^** | **Variant ID** | **Alleles^b^** | **Variant class** | **CMI / 1KG MAF^c^** | **Follow-up^d^** | **All affecteds^e,f^** | **Reduced penetrance^f^** |
| --- | --- | --- | --- | --- | --- | --- | --- | --- | --- |
| GDF6 | 8 | 97154593 | NA | T/G | 3' UTR | 0.005 / NA | Yes | No (1/2) | Yes (1/2) |
| GDF6 | 8 | 97154790 | rs36028712 | T/C | 3' UTR | 0.063 / 0.066 | No |  |  |
| GDF6 | 8 | 97154813 | rs112542818 | C/T | 3' UTR | 0.026 / 0.003 | Yes | No (5/8) | Yes (2/4) |
| GDF6 | 8 | 97155360-97155362 | rs139734303 | CCT/C | 3' UTR | 0.010 / 0.02 | No |  |  |
| GDF6 | 8 | 97155701 | rs80085212 | C/G | 3' UTR | 0.010 / 0.017 | No |  |  |
| GDF6 | 8 | 97155707 | rs77181285 | G/T | 3' UTR | 0.010 / 0.017 | No |  |  |
| GDF6 | 8 | 97155793 | rs114201878 | C/G | 3' UTR | 0.010 / 0.017 | No |  |  |
| GDF6 | 8 | 97156214 | rs75587676 | T/A | 3' UTR | 0.010 / 0.017 | No |  |  |
| GDF6 | 8 | 97156411 | rs2440199 | T/C | 3' UTR | 0.599 / 0.414 | No |  |  |
| GDF6 | 8 | 97156472 | rs79053711 | T/G | 3' UTR | 0.005 / 0.02 | No |  |  |
| GDF6 | 8 | 97157223 | rs148861809 | C/G | Coding-syn | 0.036 / 0.028 | Yes | No (7/10) | Yes (3/8) |
| GDF6 | 8 | 97157413 | rs121909352 | G/T | Missense | 0.016 / 0.003^g^ | Yes | No (4/6)^h^ | Unknown (0/3) |
| GDF6 | 8 | 97157791 | rs77859767 | G/C | Intronic | 0.005 / NA | No |  |  |
| GDF6 | 8 | 97157792 | rs11783820 | G/C | Intronic | 0.010 / 0.053 | No |  |  |
| GDF6 | 8 | 97157811 | NA | T/A | Intronic | 0.005 / NA | Yes | No (1/2) | Yes (1/2) |
| GDF6 | 8 | 97158024-97158030 | NA | C/CGGCGGC | Intronic | 0.005 / NA | No |  |  |
| GDF6 | 8 | 97159015 | NA | A/G | Intronic | 0.005 / NA | No |  |  |
| GDF6 | 8 | 97159446-97159447 | NA | T/TA | Intronic | 0.005 / NA | No |  |  |
| GDF6 | 8 | 97159651 | rs76595269 | C/T | Intronic | 0.099 / 0.123 | No |  |  |
| GDF6 | 8 | 97169735 | NA | C/T | Intronic | 0.010 / NA | Yes | Yes (2/2) | Yes (1/2) |
| GDF6 | 8 | 97169759 | rs116719470 | C/T | Intronic | 0.021 / 0.049 | No |  |  |
| GDF6 | 8 | 97169811 | rs7845829 | G/C | Intronic | 0.089 / 0.09 | No |  |  |
| GDF6 | 8 | 97170374 | rs140757891 | C/T | Intronic | 0.021 / 0.013 | Yes | Yes (4/4) | Yes (2/10) |
| GDF6 | 8 | 97172403 | rs2466129 | A/G | Intronic | 0.495 / 0.474 | No |  |  |
| GDF6 | 8 | 97172487 | rs62516290 | G/T | Intronic | 0.078 / 0.077 | No |  |  |
| GDF3 | 12 | 7842587 | rs2302516 | C/G | Missense | 0.047 / 0.024 | Yes | No (7/11) | Yes (1/14) |
| GDF3 | 12 | 7842932 | rs12819884 | C/T | Missense | 0.353 / 0.339 | No |  |  |
| GDF3 | 12 | 7848202 | rs17727707 | G/C | Coding-syn | 0.067 / 0.075 | No |  |  |

^a^Base pair positions based on human genome build GRCh37/hg19

^b^Alleles: Reference allele / Alternate allele

^c^CMI MAF estimate based on all affected family members initially screened; 1KG MAF: Based on 1000 Genomes Integrated Phase 1 Release v3: European population

^d^Variant was validated by bidirectional sequencing and all sampled affected and unaffected individuals within each identified family were sequenced

^e^Is sharing observed across all affected individuals within each family?

^f^Numbers in parentheses: Numerator: number of sampled individuals carrying the variant, Denominator: total number of sampled individuals

^g^MAF estimate was not available from 1000 Genomes; MAF estimate based on the Exome sequencing project: European population

^h^Individual diagnosed with Chiari Malformation Type 0 is counted as "affected" for the purposes of this table

Abbreviations: Chr: Chromosome; Coding-syn: Coding-synonymous; MAF: Minor allele frequency
